# Notes and Notices

**Published:** 1885-11

**Authors:** 


					﻿NOTES AND NOTICES.
Fob, Sale.—We have two complete sets (5 volumes) of this journal for
sale.
The North American Journal of Homceopathy, Volume I, No. 1
(the third series), under new management, is just out. The initial number
presents a neat appearance and is well supplied with good matter. The
Journal will hereafter be issued monthly at three dollars per annum, will be
conducted by a corps of editors, viz.: Drs. George M. Dillon, Sidney F. Wil-
cox, Malcolm Seal, Clarence E. Beebe, Charles E. Sterling, and Eugene H.
Porter. Dr. George G. Shelton is to be the manager, and the office at 10
East Thirty-sixth Street, New York City.
Germs in the Air.—M. de Parville has published a paper on 'the pre-
sence of bacteria in the air we breathe. He says that the proportion of bac-
teria in a cubic meter is 6 in sea air, 1 in the air of high mountains, 60 in the
principal cabin of a ship at sea, 200 in the air at the top of the Pantheon in
Paris, 360 in the Rue de Rivoli of Paris, 6,000 in the Parisian sewers, 36,000
in old Parisian houses, 40,000 in the new hospital of the Hotel Dieu of Paris,
and 79,000 in the old hospital of Pitie, in Paris. In Ryder street, St. James,
London, a cubic meter of air contains only 240 bacteria.
				

